# MicroRNA-138 Suppresses Neutrophil Gelatinase-Associated Lipocalin Expression and Inhibits Tumorigenicity

**DOI:** 10.1371/journal.pone.0052979

**Published:** 2012-12-31

**Authors:** Ying Chu Lee, Woan-Fang Tzeng, Tzeon-Jye Chiou, Sin Tak Chu

**Affiliations:** 1 Institute of Biological Chemistry, Academia Sinica, Taipei, Taiwan; 2 Department of Life Sciences, Fu Jen University, Taipei, Taiwan; 3 Division of Transfusion Medicine, Department of Medicine, Taipei Veterans General Hospital, Taipei, Taiwan; 4 National Yang-Ming University School of Medicine, Taipei, Taiwan; 5 Institute of Biochemical Sciences, College of Life Science, National Taiwan University, Taipei, Taiwan; University of Illinois College of Medicine, United States of America

## Abstract

The expression of neutrophil gelatinase-associated lipocalin (NGAL) is up-regulated in some cancers; therefore NGAL has potential as a tumor biomarker. Although the regulation mechanism for this is unknown, one study has shown that it is likely to involve a microRNA (miRNA). Here, we investigate the relation between miRNA expression and NGAL expression, and the role of NGAL in tumorigenesis. Using miRNA target–detecting software, we analyze the mRNA sequence of NGAL and identify a target site for microRNA-138 (miR-138) in nucleotides 25–53 of the 3′ UTR. We then analyze NGAL and miR-138 expression in three cancer cell lines originating from breast, endometrial and pancreatic carcinomas (the MCF-7, RL95-2 and AsPC-1 cell lines), respectively, using quantitative (real-time) PCR and western blot analysis. Metastasis is a critical event in cancer progression, in which malignant cell proliferation, migration and invasion increase. To determine whether miR-138-regulated NGAL expression is associated with metastasis, the proliferation and migration of the cell line are examined after miR-138 transfection. Using nude mice, we examine both the tumorigenicity of these cell lines and of miR-138-transfected cancer cells *in vivo*, as well as the effect of treating tumors with an antibody against NGAL. Our results show that these cancer cell lines down-regulate NGAL when miR-138 is highly expressed. Ectopic transfection of miR-138 suppresses NGAL expression and cell migration in RL95-2 and AsPC-1 cells, demonstrating that miR-138-regulated NGAL expression is associated with cell migration. Additionally, injection of the NGAL antibody diminishes NGAL-mediated tumorigenesis in nude mice, and miR-138 transfection of cancer cells reduces tumor formation. As the cell proliferation data showed that the tumor size should be regulated by NGAL-related cell growth. Taken together, our results indicate that NGAL may be a good target for cancer therapy and suggest that miR-138 acts as a tumor suppressor and may prevent metastasis.

## Introduction

Neutrophil gelatinase-associated lipocalin (NGAL) was first isolated as a novel 25 kDa protein complex associated with human neutrophil gelatinase [1]. Under various physiological conditions leading to its increased expression, NGAL has been identified as a survival factor (or apoptotic factor); however, the mechanism by which this occurs is unknown [2], [3]. NGAL has been detected in a number of tumor types, including breast, pancreatic and colon cancer, and it has been suggested that it could be used as a biomarker for cancer [4]–[6]. Leng et al. [7] observed that the reduction of NGAL expression in breast cancer cells by injecting an antibody for NGAL inhibited tissue invasion by breast cancer cells and their migration. Down-regulation of NGAL expression in certain cancer cells or use of antibody neutralization against NGAL may, therefore, be effective in reducing metastasis and possibly provide a potential therapeutic approach for cancer.

MicroRNAs (miRNAs) regulate various biological functions via post-transcriptional gene silencing. Their importance in the regulation of gene expression and, thus, protein expression is correlated with disease formation in many organisms, as abnormal miRNA levels trigger gene up- or down- regulation in many cancers [8]. Using computational approaches, targets of miRNA can be predicted in 3′ untranslated regions (3′ UTRs) of transcripts, *i.e.,* the miRNA nucleotide fragment binds to a 3′ UTR sequence and suppresses translation [9], [10]. Because miRNA-suppressed genes may be involved in complex diseases or cancers, these small molecules could potentially be developed as diagnostic markers and therapeutic targets.

The up-regulation of NGAL expression has been implicated in ovarian cancer, and NGAL may play a role in the epithelial-mesenchymal transition via epidermal growth factor induction [11]. The epithelial to mesenchymal transition (EMT) is characterized by the transformation of epithelial cells to migratory mesenchymal cells and is an important event in tumorigenesis [12]. The level of NGAL is also reflected by the cancer grade and stage [11]. Liu et al. [13] have shown that miRNA-138 (miR-138) suppresses an EMT in squamous carcinoma cell lines and regulates cell migration and invasion. In a recent study in our laboratory (unpublished data), we demonstrated that up-regulation of NGAL in endometrial epithelial cells triggered an EMT under stress condition. This finding led to our investigation of the role of miR-138, a multi-functional molecular regulator which may regulate NGAL expression and thereby play a role in NGAL-induced tumorigenesis.

## Materials and Methods

### Animals

Eight-week-old BALB/c nude mice (BALB/cAnN.*Cg-Foxn1nu*/*C­rlNarl*) were purchased from the National Laboratory Animal Center (Taipei, Taiwan) and bred in a specific pathogen-free room in the animal facility. Permission for the nude mice animal study was obtained from the Institutional Animal Care and Use Committee (IACUC) of the National Laboratory Animal Center (Permit Number IACUC2012-037). This study was carried out in strict accordance with the recommendations in the Guide for the Care and Use of Laboratory Animals of the National Institutes of Health. All surgeries were performed under sodium pentobarbital anesthesia, and all efforts were made to minimize suffering.

### Cell Lines

All the cell lines, including: HEK293T cells, HCT116, HeLa, OVCAR-3, human pancreatic and breast adenocarcinoma cells (AsPC-1 and MCF-7 cells), respectively, and uterine endometrial carcinoma cells (RL95-2) were obtained from American Type Culture Collection (Rockville, MD). AsPC-1 cells were cultured in RPMI-1640 medium (HyClone, Thermo Scientific, USA) supplemented with fetal bovine serum (FBS) (HyClone, Thermo Scientific, USA) to obtain a final concentration of 10% FBS. MCF-7 cells were cultured in 90% [Minimum Essential Medium with Earle’s Balanced Salts (-Minimum Essential Medium; HyClone Classical Powdered Media, Thermo Scientific, Waltham, MA)], supplemented with FBS and non-essential amino acids (Gibco, Invitrogen, Carlsbad, CA) to obtain final concentrations of 10% and 0.1 mM, respectively. RL95-2 cells were cultured in DMEM/F12 medium (HyClone, Thermo Scientific, USA) supplemented with FBS and insulin to obtain final concentrations of 10% and 5 µg/ml, respectively. All the cells were cultured at 37°C in a humidified atmosphere of 5% CO_2_ in air.

### The mcroirna Prediction

The microRNA candidates targeted to the NGAL-3′ UTR sequence were identified by cross-matching the results of searching for target sites of known microRNAs using four different websites: the microRNA.org resource (www.microRNA.org), the miRanda algorithm implemented at the miRBase database (www.mirbase.org), miRDB (www.mirdb.org) and TargetScanHuman v5.1 (www.targetscan.org) [14].

### TaqMan miRNA Assay

Total RNA was isolated from AsPC-1, MCF-7 and RL95-2 cell pellets using a miRNeasy Mini kit (Qiagen, Hilden, Germany) according to the manufacturer’s protocol. For miRNA cDNA synthesis, RNA was reverse transcribed using the TaqMan MicroRNA Reverse Transcriptase kit (Applied Biosystems, Foster City, CA) according to the manufacturer’s protocol. PCR reactions were performed using a 7300 Real-Time PCR System and a TaqMan MicroRNA Assay (Applied Biosystems) as the reaction mixture. The PCR cycling conditions used were 50°C for 2 min and 95°C for 10 min, followed by 40 cycles of 95°C for 15 sec and 60°C for 1 min. Human U6 small nuclear RNA (RUN6B) (ABI, Applied System, USA) was used as an internal control.

### Quantitative Real-time PCR (qPCR)

Total RNA from the cell lines was extracted using the RNeasy Mini kit (Qiagen) following the kit instructions. Using gene-specific primers, cDNA was synthesized from the total RNA using a High Capacity cDNA Reverse Transcription kit (Applied Biosystems) according to the manufacturer’s protocol. The cDNA product was used for the subsequent qPCR analysis, which was performed at 50°C for 2 min and 95°C for 10 min, followed by 40 cycles of 95°C for 15 sec and 60°C for 1 min. The housekeeping genes, glyceraldehyde-3-phosphate dehydrogenase (GAPDH) and β-actin, were used as internal controls. Ki-67 and TPX2 gene expression levels were used as the cell proliferation markers [15]. The primer sequences were listed: hKi-67 Fw-gaggtgtgcagaaaatccaaa; hKi-67 Rv-ctgtccctatgacttctggttgt; hTPX2 Fw-acatctgaactacgaaagcatcc; hTPX2 Rv-ggcttaacaatggtacatccctta.

### Western Blot Analysis

Western blots were performed using a SNAP i.d. Protein Detection System (Millipore, Billerica, MA) and proteins were separated using 15% SDS-PAGE gels. After blocking with a 1% w/v solution of nonfat dry milk powder in phosphate buffered saline containing 0.1% (w/v) Tween 20 for 20 sec at room temperature, NGAL protein levels were measured using a rat polyclonal NGAL antibody (R&D Systems Inc., Europe) at 1∶1,000 dilution, followed by a horseradish peroxidase-conjugated secondary antibody at 1∶4,000 dilution. Reactions were carried out at room temperature for 10 min. Gel bands were visualized by enhanced chemiluminescence according to the manufacturer’s protocol. GAPDH served as an internal loading control and was detected using monoclonal rabbit anti-GAPDH antibody (Abcam, Cambridge, UK) at 1∶4,000 dilutions.

### Cell Migration Assay

The wound-healing assay was developed to study cell migration *in vitro* by mimicking the process *in vivo* [16]. In brief, approximately 0.6–1.5 × 10^6^ cells were seeded into 12-well plates and cultured in 10% FBS containing the appropriate media until confluent. A pipette was then used to make a straight scratch to simulate a wound, and the wound area was observed by microscope over a 24 h period, until the wound had partially closed.

Using the Transwell Migration Assay, we quantified the number of migrating cells using 80 µm polyethylene terephthalate membrane cell culture inserts (Costar, Corning, MA, USA). In brief, cells were treated with the appropriate miRNA and seeded into upper chambers of the cell culture inserts. Control samples had no added miRNA. After 24 h of incubation, cells remaining in the upper chamber were removed. Cells adhering to the lower membrane were fixed with methanol and stained with 0.5% crystal violet in 20% methanol (Sigma, MO, USA) for 20 min, then rinsed twice with water. The cells on the upper surface of the filters were removed by wiping with a cotton swab. The crystal violet dye retained on the filters was then extracted with 150 µl of 33% acetic acid, and the absorbance of this solution was measured at 570 nm using an ELISA reader.

### Transfection of the AsPC-1 Cells

Transfection of the AsCP-1 cell line was carried out using Lipofectamine 2000 (Invitrogen) according to the manufacturer’s protocol. In brief, approximately 2 × 10^5^ AsPC-1 cells were transfected with lentiviral particles (HyClone, Thermo Scientific, USA) containing either miR-138 (VISMHS_000912) or a non-targeting control sequence (HMR 5872). A multiplicity of infection of the 3′ UTR was used for establishing the transfected stable lines.

### Dual-luciferase Reporter Assay

HEK293T cells were seeded in 24-well plates, and then cotransfected 0.5 µg of pGL3-NGAL-3′UTR or pGL3-NGAL-3′UTR-mut with 50 ng pRL-TK for 24 h, and then transfected with miR-138 mimic or non-targeting control RNA using Lipofectamine 2000. Cells were collected 48 h after transfection, and luciferase activity was measured using a Dual-Luciferase Reporter 1000 Assay System (Promega, Madison, WI) and recorded using a luminescence microplate reader (Packard LumiCount, Packard Instruments, Meriden, CT) according to the manufacturer’s instructions.

### *In vivo* Tumorigenicity Tests

*In vivo* tumor formation was achieved by subcutaneously inoculating tumor cells into 8-week-old nude mice. Approximately 1–5×10^6^ cells from each of the four cell types: AsPC-1, RL95-2, MCF-7 and miR-138-AsPC-1, were suspended in 50 µl of cell culture media. Five mice were used for each cell line and the mice were observed daily for up to 30 days. The group of nude mice that had been inoculated with RL95-2 cells was injected with 10 µg NGAL antibody after 14 days. Antibody injections were repeated 3 more times at 4-day intervals.

### Cell Proliferation Analysis

For analysis of cell proliferation, cells were seeded on 12-well plates at 2×10^5^cells/well and cultured for 48–72 h. Ki-67 and TPX2 gene expressions as proliferative markers were measured via real-time PCR.

### Statistical Testing

A one-way analysis of variance with a Dunnett’s test was used for statistical testing using the software InStat v 3.0 for Windows (GraphPad Software, San Diego, CA).

## Results

### Complementarity Searching

**Figure 1:**
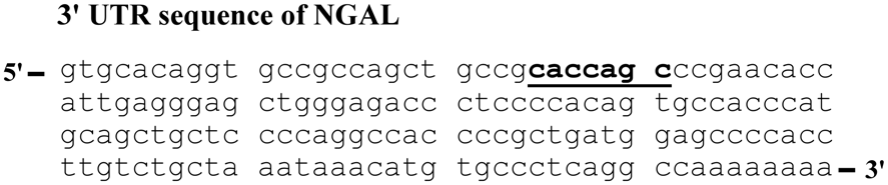
miR-138 target sequence identified in the 3′ UTR sequence of NGAL. Cross-matching results from four different miRNA target identification websites revealed the miR-138 target sequence (shown in bold, underlined) in the 3′ UTR sequence of NGAL.

We evaluated microRNA target sequences in the 3′ UTR of the NGAL transcript using four different databases. A comparison of the results showed that the microRNA target sequence (CACCAGC) of miR-138 was present in the 3′ UTR of NGAL (Fig. 1) in a higher estimated score than other microRNA, suggesting that transcriptional regulation of NGAL may be under miR-138 control. The miR-138 targeting gene was still obscure; however, this analysis showed the high possibility of the regulation in NGAL 3′ UTR. The target sequence of miR-138 (CACCAGC) was conserved in the 3′ UTR of human as well as mouse and rat. This implied that the regulation NGAL gene expression via miR-138 was important within the mammalian system.

### Expression of miRNA in Cancer Cell Lines

Because expression of miR-138 may be involved in the development of tumors, we chose six carcinoma cell lines for our preliminary investigation into the relative expression levels of NGAL mRNA and miR-138. The relative expressions levels of NGAL mRNA and miR-138 were measured for the six cell lines (Table 1). NGAL mRNA expression levels appeared to be inversely related to the expression levels of miR-138 in the cancer cell lines; thus, NGAL mRNA had high expression levels and miR-138 low expression levels in AsPC-1, RL95-2 and OVCAR-3 cells, whereas the reverse was the case for MCF-7 and HeLa cells.

**Table 1:** Relative expression levels of NGAL mRNA and miR-138 in various cancer cell lines determined by qPCR.

Cell line	HCT116	HeLa	MCF-7	AsPC-1	OVCAR-3	RL95-2
aNGAL	1	1.5	0.0004	4768.3	127	861.4
amiR-138	1	28.1	3.7	0.01	0.52	0.001

a All measurements have been normalized against miR-138 or NGAL for HCT116 cells.

**Figure 2:**
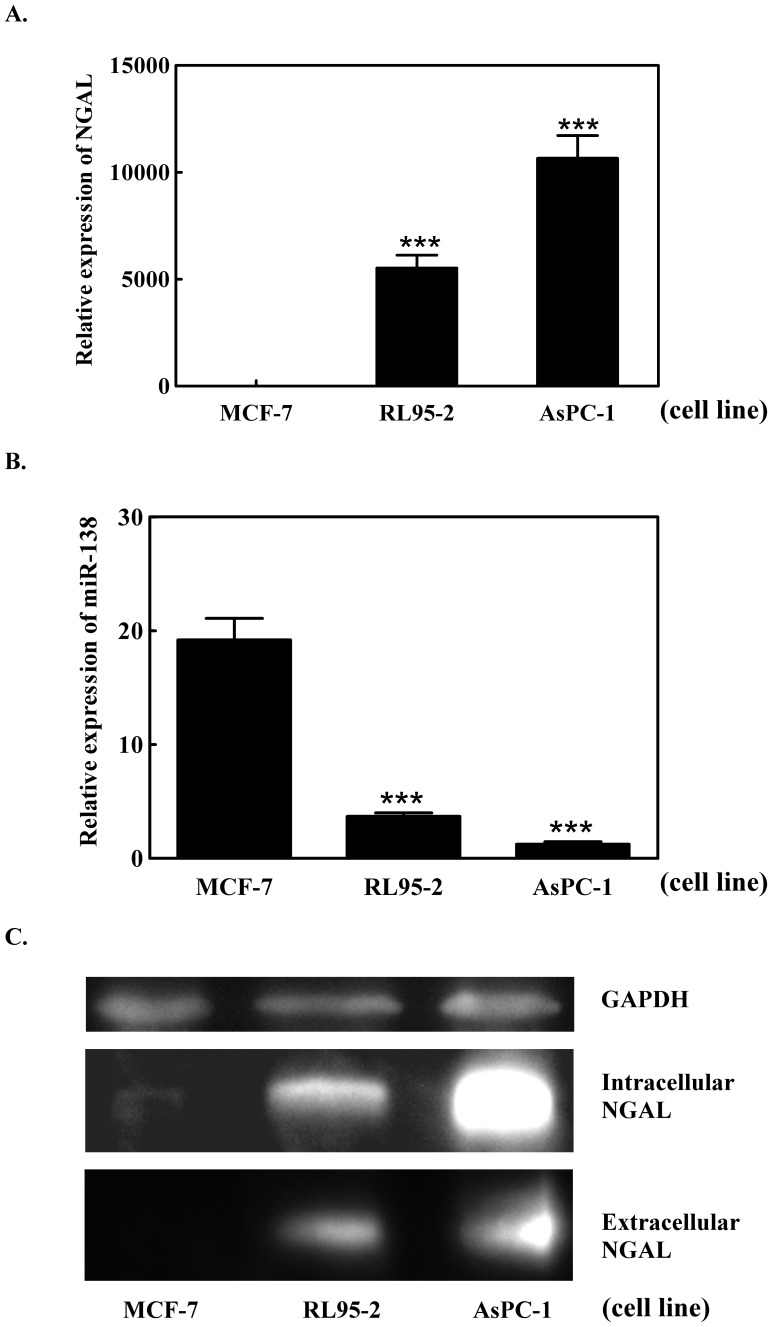
Expression of NGAL mRNA, miR-138 and the NGAL protein in three different cancer cell lines. (A) NGAL mRNA levels measured by qPCR; (B) miR-138 expression levels measured using the TaqMan MicroRNA assay; and (C) detection of intracellular and extracellular NGAL protein determined by western blot analysis in the three cancer cell lines, MCF-7, RL95-2 and AsPC-1.

MiR-138, NGAL mRNA and protein levels were measured in the three cell lines: MCF-7, AsPC-1 and RL95-2. NGAL mRNA levels were high in AsPC-1 and RL95-2 cells compared to MCF-7 cells (Fig. 2A). Levels of the intracellular and, to a lesser extent, the secreted NGAL protein product (extracellular) were also high in AsPC-1 and RL95-2 cells, but the protein could not be detected in the MCF-7 cell line (Fig. 2C). The miR-138 expression levels, however, showed the opposite behavior to NGAL mRNA expression in all three cell lines, and a high level of miR-138 was found only in the MCF-7 cell line (Fig. 2B). This indicated the existence of a relationship between miR-138 and NGAL gene expression in these cell lines.

### Effect of miR-138 on Cell Migration

**Figure 3:**
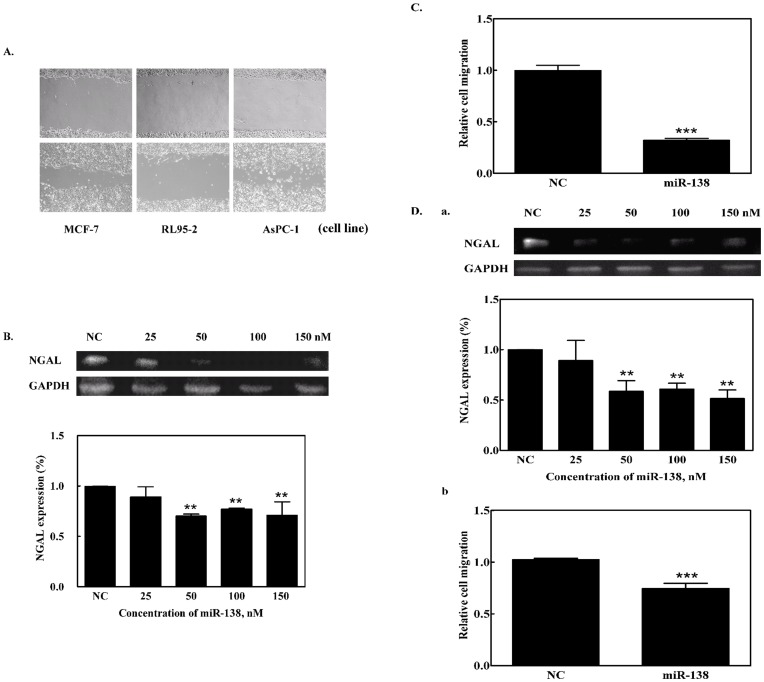
Effect of miR-138 on NGAL expression and cell migration. (A) Images of cell migration of different cell lines. Using the wound-healing assay for the three cancer cell lines, wounds were compared at 0 and 24 h after scratching. Light microscope image at 100×magnifications. (B) Western blot for NGAL expression in miR-138-transfected AsPC-1 cells. (C) For the transwell cell migration assay, cells that had migrated after a specified period were quantified using crystal violet staining. (D) The effect of miR-138 on (a) NGAL expression as determined by western blot analysis, and (b) cell migration for RL95-2 cells. ** indicates *p*<0.01, *** indicates *p*<0.001. NC: negative control, precursor miRNA.

**Figure 4:**
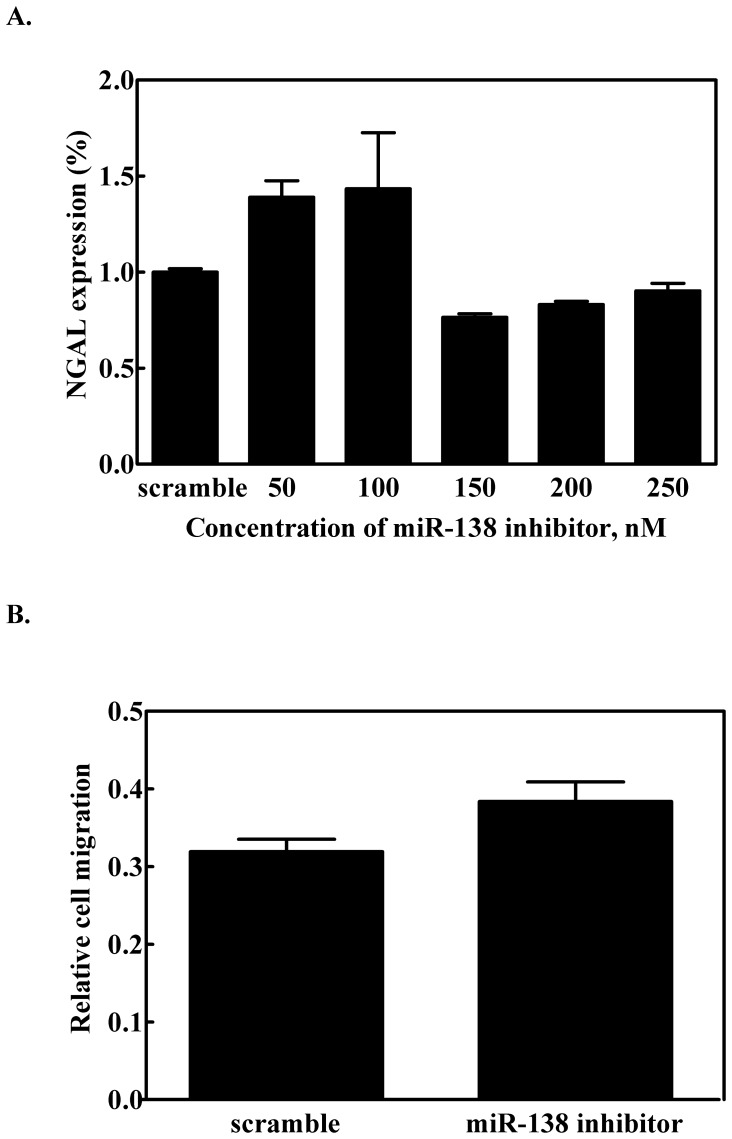
Effect of miR-138 inhibitor on NGAL expression. (A) NGAL mRNA expression levels, and (B) cell migration assay for miR-138-inhibitor transfected MCF-7 cells. Error bars indicate *p*>0.05.

Figure 3 shows the cell migration assay. Using the wound-healing method [16], the effect of miR-138 on cell migration was measured using the three cell lines over a 24 h incubation period. The RL95-2 cell line displayed noticeably slower cell migration than the other two cell types (Fig. 3A). We also examined NGAL expression in AsPC-1 cells after transfection with miR-138 and observed that transfection with 50 nM miR-138 down-regulated NGAL expression (Fig. 3B). AsPC-1 cells transfected with 100 nM miR-138 caused a reduction of NGAL expression and a concomitant slowing of cell migration (Fig. 3C). For the RL95-2 cell line, 25 nM miR-138 minimized NGAL expression (Fig. 3 D (a)); reduced cell migration was also observed when 100 nM miR-138 was transfected into RL95-2 cells (Fig. 3D (b)). For the MCF-7 cells, we transfected the cells with 100 nM miR-138 inhibitor to eliminate the effect on expression of miR-138, and then observed NGAL expression and cell migration behavior. The results showed there was no difference in the protein expression level and cell migration before and after transfection with the miR-138 inhibitor (Figs. 4A and 4B). This revealed that NGAL or miR-138 did not regulate cell migration in the MCF-7 cell line, and it hinted at the miR-138 regulation of NGAL expression shown in the cell-type specificity.

**Figure 5:**
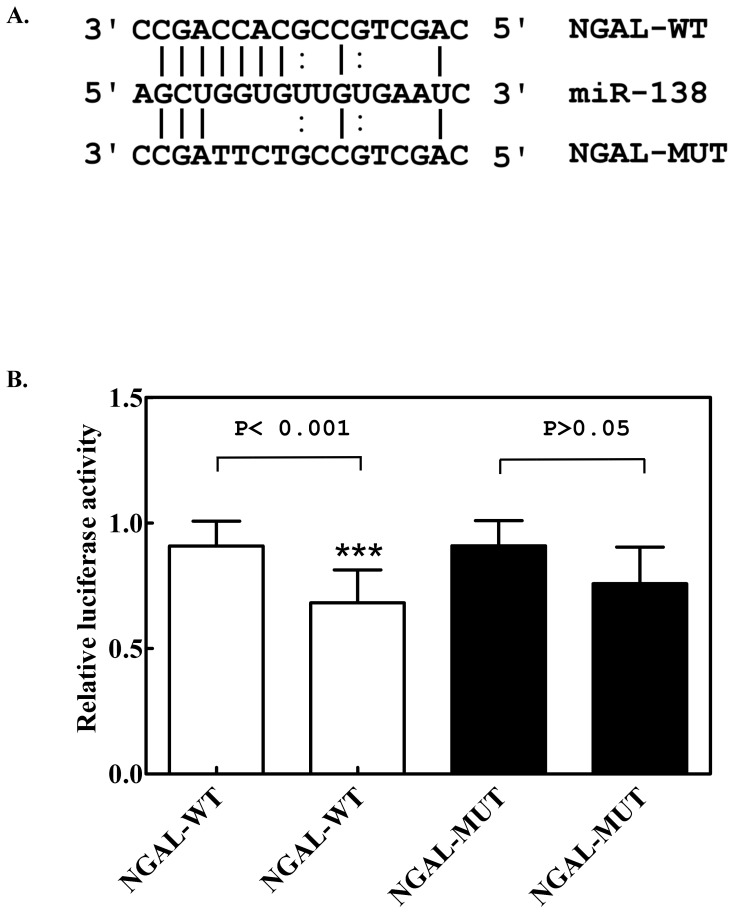
Identification of miR-138-specific target in the 3′ UTR sequence of NGAL. (A) Base pairing between miR-138 and wild-type NGAL-3′ UTR (NGAL-WT) or mutant NGAL-3′ UTR (NGAL-MUT) at the miR-138 target site. (B) Luciferase assay of HEK-293T cells transfected with a luciferase reporter gene construct that included an NGAL-3′ UTR wild-type (NGAL-WT, in white columns) or mutant (NGAL-MUT, in black columns) sequence, and then transfected with miR-138. The left column in white (or in black) indicates the control miR-transfected cells, and the right column in white (or in black) indicates the miR-138 transfected cells. Values represent mean ± SE of five experiments. NC: negative control, transfection with a random oligomer instead of miR-138. *** indicates *p*<0.001.

To further confirm target specificity between miR-138 and NGAL, we performed a luciferase reporter assay in the 293T cell line in using an NGAL 3′ UTR target site containing a downstream luciferase reporter gene. The luciferase activity of 293T cells transfected with microRNA into wild-type NGAL 3′ UTR (NGAL-WT) (Fig. 5A) was significantly lower (*p*<0.001) than that of cells transfected with mutant NGAL (NGAL-MUT), as compared to control cells (containing NGAL-WT or NGAL-MUT of 3′ UTR ) transfected with control miRNA (Fig. 5B). The results suggested that the NGAL gene was a target of miR-138, which down-regulated NGAL protein expression via post-translational modification.

### Cancer Cell Lines form Tumors in Nude Mice

**Figure 6:**
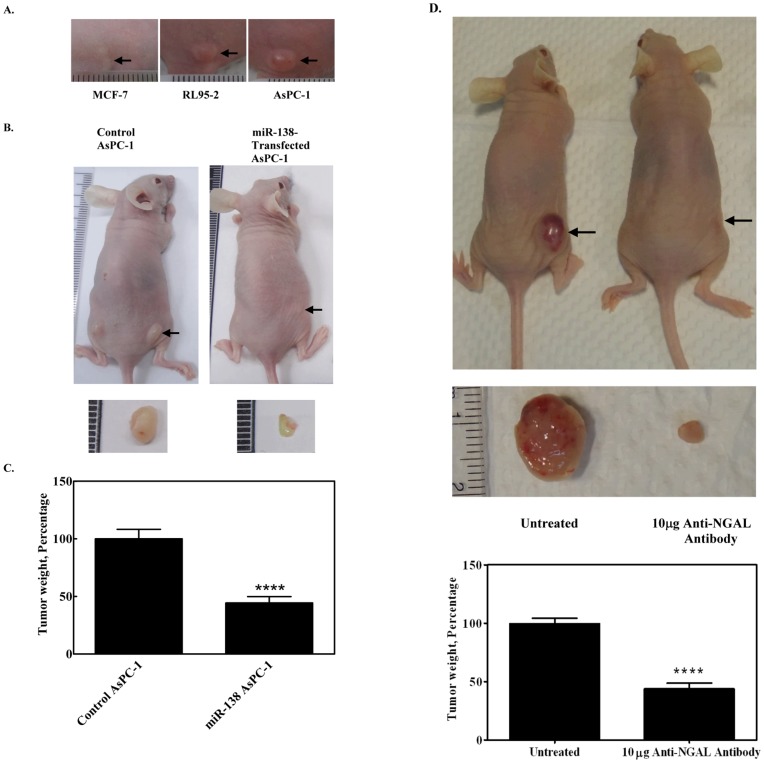
*In vivo* tumorigenicity test. (A) Tumors were formed after 30 days in nude mice after inoculation with three different cell lines (using 5 × 10^6^ cells/mouse). (B) Comparison of tumorigenicity of 1 × 10^6^ AsPC-1 cells stably transfected with Lentiviral-miR-138 or Lentiviral-non-targeting control in nude mice. Size of excised tumor is shown below. (C) Comparison of the tumor weight of the AsPC-1-inoculated mice 10 days after inoculation with miR-138-transfected or non-targeting control-transfected nude mice. **** indicates *p*<0.0001; arrows indicate location of tumors. (D) Effect of NGAL antibody on tumorigenicity *in vivo*. Tumors were formed in nude mice by inoculating with 5×10^6^ RL95-2 cells. After 14 days, NGAL antibody was injected. Typical results 30 days after the initial inoculation are shown: left, control: no antibody injected; right, NGAL antibody injected as a control. Arrows indicate position of the tumor. Size of excised tumor is shown in lower panel.

To investigate whether the cell lines induced tumor formation, nude mice were inoculated with each of the three cell types (5×10^6^ cells/mouse) and monitored over a period of time. Tumors were found in nude mice inoculated with AsPC-1 and RL95-2, but not in those inoculated with MCF-7 (Fig. 6A). Since NGAL is highly expressed in AsPC-1 cells, and is related to tumorigenesis, we investigated the effect of miR-138 on the tumorigenesis of AsPC-1 cells in nude mice. Two groups of nude mice (five mice per group) were inoculated with AsPC-1 cells and miR-138-transfected AsPC-1 cells (1×10^6^ cells/mouse). Tumors appeared after 10 days: representative photographs of the mice with tumors are shown in Fig. 6B, and the average relative tumor weight for each group in Fig. 6C. Tumorigenicity was clearly reduced (by more than 50%; *p*<0.001) for the mice inoculated with miR-138-transfected AsPC-1 cells. This suggested that the presence of miR-138 had a marked effect on tumorigenicity. If NGAL expression were related to tumorigenesis, elimination of NGAL would be expected to further reduce tumor formation. We investigated this by monitoring tumor size in nude mice that had been inoculated with RL95-2 cells and then treated 14 days later with NGAL antibody (10 µg NGAL antibody injected on days 14, 17, 20 and 23). Typical resulting tumors appeared greatly reduced in size after treatment with the NGAL antibody (Fig. 6D), suggesting that NGAL was indeed involved in tumor formation.

**Figure 7:**
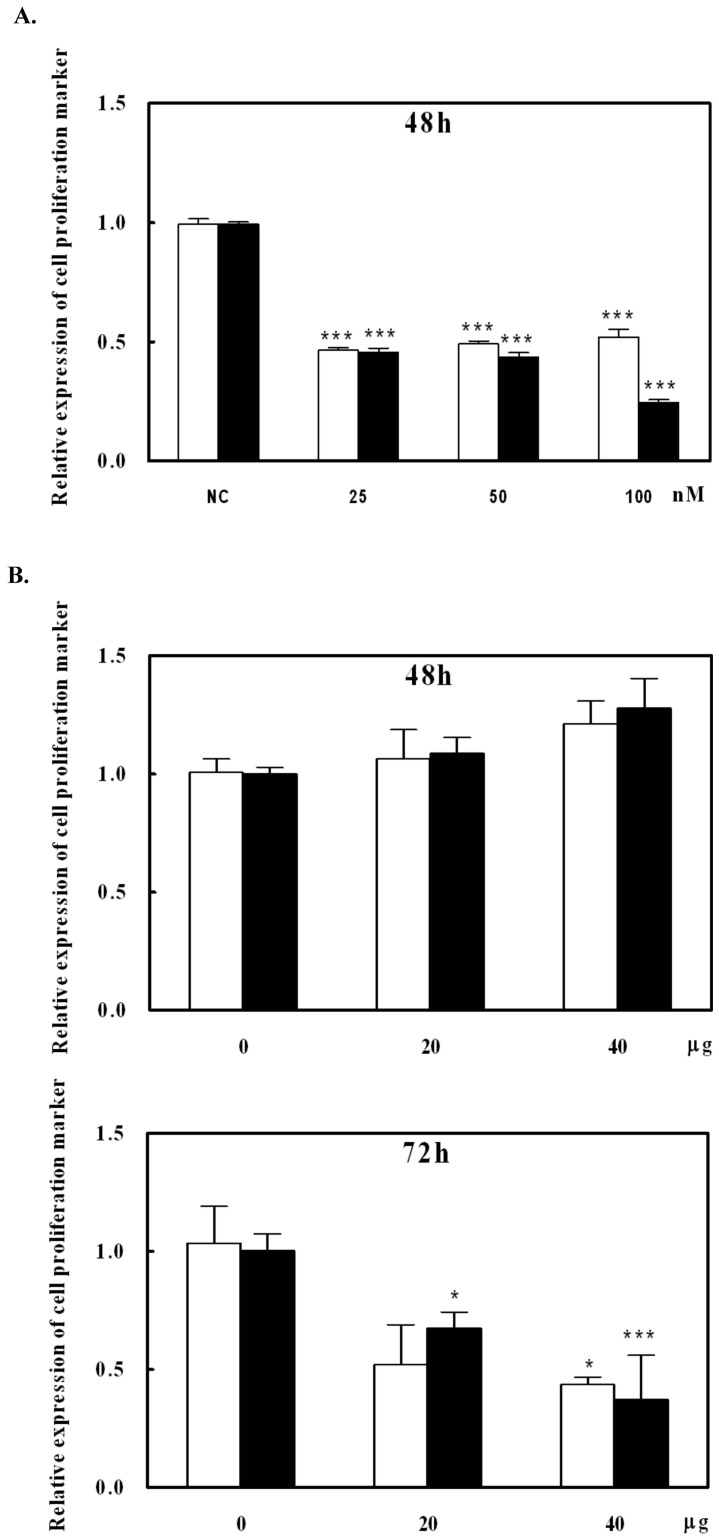
Effect of NGAL on cell growth *in vitro*. Using AsPC-1 cells as a testing cell line, Ki-67 (white bars) and TPX2 (black bars) gene expression levels were measured. (A) After miR-138 transfection to AsPC-1 cells for 48 h, total RNA was collected and two gene expressions were measured. (B) After NGAL antibody supplement for 48 and 72 h to the cultured cells, total RNA was collected and two gene expressions were measured. * and *** indicate *p*<0.5 and p<0.001, respectively.

Reduction of tumor size should be related to cell proliferation. To investigate whether the miR-138 and NGAL antibody affected cell proliferation, cell growth was assessed via a carcinoma cell line (AsPC-1). Gene expression levels of two genes: Ki-67 and TPX2 [15], were measured by real-time PCR after the cell treatment. After miR-138 transfection to AsPC-1 cells for 48 h, Ki-67 and TPX2 gene expression levels were significantly reduced (Fig. 7A, *p*<0.001). This indicated that the suppression of NGAL gene expression inhibited the AsPC-1 cell growth. Interestingly, under 20 or 40 µg NGAL antibody-supplemented media, the inhibition of cell growth needed a longer period to suppress the Ki-67 and TPX2 gene expressions (Fig. 7B). This suggested that miR-138 suppression of NGAL expression might be more effective than NGAL antibody treatment in reducing tumor growth.

## Discussion

A number of lipocalins, including NGAL, are expressed at high levels during tumorigenesis [17]. NGAL also acts as a survival factor to increase cell proliferation under some circumstances [3]. Based on our data (Fig. 2C), NGAL was found as a secreted protein, indicating that it may also play an extracellular role in tumorigenicity. Both of the data of miR-138 transfection and antibody neutralization showed the suppression of the cell growth (Fig. 7) could explain the observation that when NGAL was neutralized by its antibody, tumorigenicity was diminished (Fig. 6D). NGAL has been found in human breast cancer, ovarian cancer, colorectal cancer, pancreatic cancer and hepatic cancer [18]–[20]. We found that different tumor-derived cancer cell lines exhibited different expression levels of NGAL, which suggested that NGAL is elevated at different stages of cancer [19]. Apart from HCT-116 cells where there was no obvious correlation, the other five cell lines examined showed high levels of NGAL occurring with low levels of miR-138 (for example in AsPC-1 cells) or vice versa. Furthermore, transfection of miR-138 into AsPC-1 and RL95-2 cell lines suppressed NGAL expression, indicating that miR-138 contributed to the regulation of NGAL expression in cancer cells; it has been suggested that this may occur in a tissue-specific manner [21]. Our study has shown that NGAL expression and cell migration capability were lowered in AsPC-1 and RL95-2 cells after miR-138 transfection, suggesting that NGAL suppression may result in the loss of the ability to undergo epithelial-mesenchymal transition, a step that is essential for malignancy in tumor metastasis [19]. Liu et al. [22] have shown that the down-regulation of miR-138 in a carcinoma squamous cell line is associated with the enhancement of cell migration via mesenchymal transition. It has also been suggested that NGAL promotes cell motility in colon carcinoma cells [6]. In Wang’s report has mentioned that microRNA inhibits breast cancer cell proliferation and suggested microRNA may be a tool for breast cancer therapy [23]. The data of figure 7 has provided the evidence for the reduction of tumor size via inhibition of cell proliferation resulting from the NGAL protein amount in the microenvironment. We might have the same suggestion. NGAL expression may, therefore, be associated with tumorigenesis, and the phenomenon eliminated by miR-138 and NGAL antibody. Of course, we could not exclude miR-138 from the regulation of other genes to slow down cell proliferation. However, according to the results of our tumorigenicity tests, NGAL was a contributor to oncogenesis in some cancers and increased the risk of metastasis; it may, therefore, be a valuable target for therapeutic intervention in some cancers.
